# Re-Invasion of H5N8 High Pathogenicity Avian Influenza Virus Clade 2.3.4.4b in Hokkaido, Japan, 2020

**DOI:** 10.3390/v12121439

**Published:** 2020-12-14

**Authors:** Norikazu Isoda, Augustin T. Twabela, Enkhbold Bazarragchaa, Kohei Ogasawara, Hirotaka Hayashi, Zu-Jyun Wang, Daiki Kobayashi, Yukiko Watanabe, Keisuke Saito, Hiroshi Kida, Yoshihiro Sakoda

**Affiliations:** 1 Laboratory of Microbiology, Department of Disease Control, Faculty of Veterinary Medicine, Hokkaido University, Sapporo 060-0818, Hokkaido, Japan; nisoda@vetmed.hokudai.ac.jp (N.I.); ttaugushahu@vetmed.hokudai.ac.jp (A.T.T.); bazarragchaa@vetmed.hokudai.ac.jp (E.B.); hayashihirotaka@vetmed.hokudai.ac.jp (H.H.); wangzujyun@vetmed.hokudai.ac.jp (Z.-J.W.); kobayashi-dk@eis.hokudai.ac.jp (D.K.); 2 International Collaboration Unit, Research Center for Zoonosis Control, Hokkaido University, Sapporo 001-0020, Hokkaido, Japan; kida@vetmed.hokudai.ac.jp; 3 Institute for Raptor Biomedicine Japan Co., Ltd., Kushiro 084-0922, Hokkaido, Japan; k_ogasawara@irbj.net (K.O.); y_watanabe@irbj.net (Y.W.); k_saito@irbj.net (K.S.); 4 Research Center for Zoonosis Control, Hokkaido University, Sapporo 001-0020, Hokkaido, Japan

**Keywords:** highly pathogenic avian influenza, clade 2.3.4.4, H5N8

## Abstract

Global dispersion of high pathogenicity avian influenza (HPAI), especially that caused by H5 clade 2.3.4.4, has threatened poultry industries and, potentially, human health. An HPAI virus, A/northern pintail/Hokkaido/M13/2020 (H5N8) (NP/Hok/20) belonging to clade 2.3.4.4b, was isolated from a fecal sample collected at a lake in Hokkaido, Japan where migratory birds rested, October 2020. In the phylogenetic trees of all eight gene segments, NP/Hok/20 fell into in the cluster of European isolates in 2020, but was distinct from the isolates in eastern Asia and Europe during the winter season of 2017–2018. The antigenic cartography indicates that the antigenicity of NP/Hok/20 was almost the same as that of previous isolates of H5 clade 2.3.4.4b, whereas the antigenic distances from NP/Hok/20 to the representative strains in clade 2.3.4.4e and to a strain in 2.3.4 were apparently distant. These data imply that HPAI virus clade 2.3.4.4b should have been delivered by bird migration despite the intercontinental distance, although it was not defined whether NP/Hok/20 was transported from Europe via Siberia where migratory birds nest in the summer season. Given the probability of perpetuation of transmission in the northern territory, periodic updates of intensive surveys on avian influenza at the global level are essential to prepare for future outbreaks of the HPAI virus.

## 1. Introduction 

Avian influenza is a highly contagious disease that is caused by infection with influenza A viruses, categorized into highly or low pathogenic influenza viruses according to its pathogenicity in chickens [1]. Influenza A viruses originating from wild birds are subtyped by reactivity to antisera against two glycoproteins, hemagglutinin (HA) and neuraminidase (NA), on the virus surface, to H1-16 and N1-9, respectively [2]. High pathogenicity avian influenza (HPAI) viruses are limited to either the H5 or H7 subtypes and possess multiple basic amino acid residues at the site of HA proteolytic cleavage, leading to a systemic infection with ubiquitous protease in the avian hosts [3].

In 1997, a large-scale HPAI outbreak occurred in Hong Kong leading to 18 human infections with 3 deaths and culling of 1.2 million poultry [4]. The causal agent of the 1997 HPAI outbreak was the H5N1 HPAI virus (HPAIV), which originated from the prevalent strain in domestic geese of Guangdong Province, China, in 1996 [5]. Following continuous HPAI outbreaks in Asia, the introduction of H5N1 HPAIV by migratory birds, which are natural reservoirs of the avian influenza virus, could contribute to an interregional spread of infectious viruses [6]. Through the worldwide spread of H5 HPAIV, the H5 HA strains have evolved from an isolated strain in Guangdong, 1996, into 10 genetically distinct virus clades and multiple subclades [7], in which the antigenicity of H5 HPAIVs has varied accordingly [8,9]. In particular, the emergence of HPAIV H5 HA clade 2.3.4.4, which evolved from the prevalent strain within China (clade 2.3.4) was recognized not only in eastern Asia due to genetic reassortment of other H5 HPAIV clades and local low pathogenic avian influenza viruses (LPAIV), but also in western Asia, Europe, and North America [10,11,12,13,14,15,16,17]. As of end of 2019, nomenclature of the H5 HA was updated to divide the group a to h due to further genetic evolvement confirmed among the recent isolates [18].

Circulation of various groups of HPAIV necessitates an intensive surveillance of avian influenza virus among wild and migratory birds to provide an update on the virus circulation situation so that proper measures can be implemented to mitigate its invasion. In October 2020, we isolated an HPAIV H5N8 clade 2.3.4.4b from a fecal sample of a migratory bird that usually nests in the northern territory, collected at Lake Komuke in the northeastern part of Hokkaido, Japan. The objective of the present study was to characterize the genetic and antigenic properties of the novel isolate of A/northern pintail/Hokkaido/M13/2020 (H5N8) (NP/Hok/20). 

## 2. Materials and Methods 

**Figure 1:**
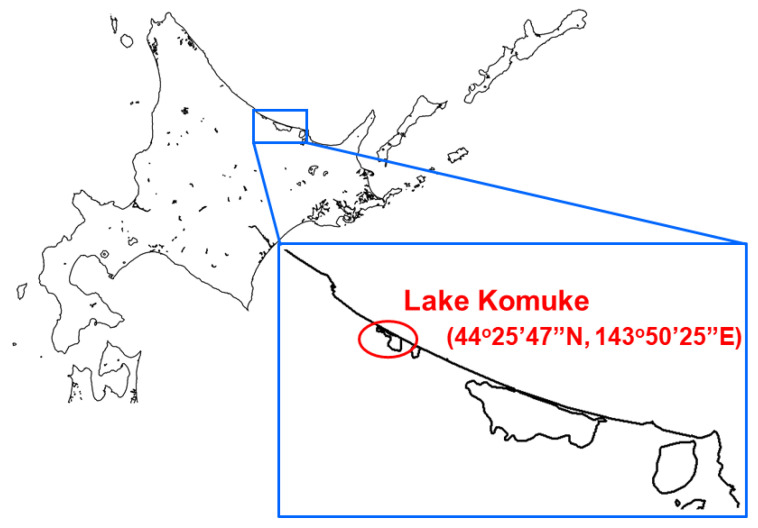
Location of a fecal sample collection. Fecal samples were collected at Lake Komuke, northeastern Hokkaido, in Japan in October 2020.

A total of 100 duck fecal samples were collected at the lakeside of Lake Komuke (latitude: 44°25′47″ north; longitude: 143°50′25″ east) in the eastern part of Hokkaido, Japan on 24 October 2020 (Figure 1). Each of the fecal samples mixed with Minimum Essential Medium (Nissui, Tokyo, Japan) containing antibiotics was inoculated into 10-day-old embryonated chicken eggs for virus isolation [19]. After virus propagation in the allantoic fluid, the influenza virus subtype was determined by hemagglutination inhibition (HI) and neuraminidase inhibition (NI) tests using antisera against the referenced influenza virus strain for subtyping [20].

DNA was extracted from the fecal sample using a DNA Mini Kit (Qiagen, Hilden, Germany), according to the manual instructions. A 749 bp region near the 5’ terminus of the cytochrome *c* oxidase I (COI) gene of a host bird was amplified using primers described in the previous study [21]. DNA sequences of the products of polymerase chain reaction were compared with the sequence data of the COI gene obtained from the Barcode of Life Data Systems (http://www.barcodinglife.com).

Viral RNA was extracted from the allantoic fluid of embryonated chicken eggs using TRIzol LS Reagent (Life Technologies, Carlsbad, CA, USA), and then was amplified by PCR reaction using each of 8 segment gene-specific primer sets for each of the 8 segments after reverse transcription [22]. Direct sequencing of each gene was performed using BigDye Terminator version 3.1, a Cycle Sequencing Kit (Life Technologies), and a 3500 Genetic Analyzer (Life Technologies). The nucleotide sequence datasets of 8 gene segments from the representative H5N8 clade 2.3.4.4 strains, or HxN8, including outgroup viruses downloaded from the Global Initiative on Sharing All Influenza and GenBank, were prepared with adequate alignment for constructing phylogenetic trees. The maximum-likelihood method was applied to construct the phylogenetic tree of each gene segment using the best-fit general time-reversible model of the nucleotide substitution with gamma-distribution rate variation among sites (with four rate categories, Γ) in MEGA 10 software [23]. The definition of the H5 HA clade in the present study followed the update of nomenclature for phylogenetic relationships, which had been scheduled at the end of 2019 [18].

Cross-reactivity of the antisera and their corresponding antigens was assessed using the HI test (Table S1). The A/chicken/Kumamoto/1-7/2014 (H5N8) (Ck/Kum/1-7/14) was isolated from a chicken in Japan. The A/Muscovy duck/DR Congo/KAF1/2017 (H5N8) (MDk/CD/KAF1/17) was isolated from a Muscovy duck in DR Congo. The A/black swan/Akita/1/2016 (H5N6) (BS/Aki/1/16) was isolated from a black swan in Japan. The A/duck/Vietnam/HU1-1151/2016 (H5N6) (Dk/VTN/1151/16) was isolated from a Pekin duck in Vietnam. The A/peregrine falcon/Hong Kong/810/2009 (H5N1) (PF/HK/810/09) was isolated from a peregrine falcon in Hong Kong. We also included in the panel antiserum against A/mallard duck/Hokkaido/24/2009 (H5N1) (Mal/Hok/24/09), which is an unclassified LPAIV isolated from wild birds in Japan [19]. An antigenic cartograph based on the results of the HI test was estimated using web-based software [24]. The data of the results of cross-HI titers were loaded to obtain the x/y coordinates of each antiserum and antigen.

## 3. Results

Among the 100 fecal samples collected at Lake Komuke, two influenza A viruses were isolated, one of which was H14N2, the other was subtyped as H5N8 by the HI and NI tests and designated as A/northern pintail/Hokkaido/M13/2020 (H5N8) (NP/Hok/20). The sequence analyses revealed that the HA of NP/Hok/20 had multiple basic amino residues at the proteolytic cleavage site, REKRRKR/G, which is defined as a molecular marker of HPAIV [25]. In the COI gene database, the sequence of mitochondrial DNA extracted from the fecal sample showed 100% homology to the COI gene of *Anas acuta* (northern pintail). The embryonated chicken eggs and two chickens intranasally inoculated with NP/Hok/20 died within 48 to 72 h post-inoculation, respectively (data not shown), suggesting that NP/Hok/20 is an HPAIV.

**Figure 2:**
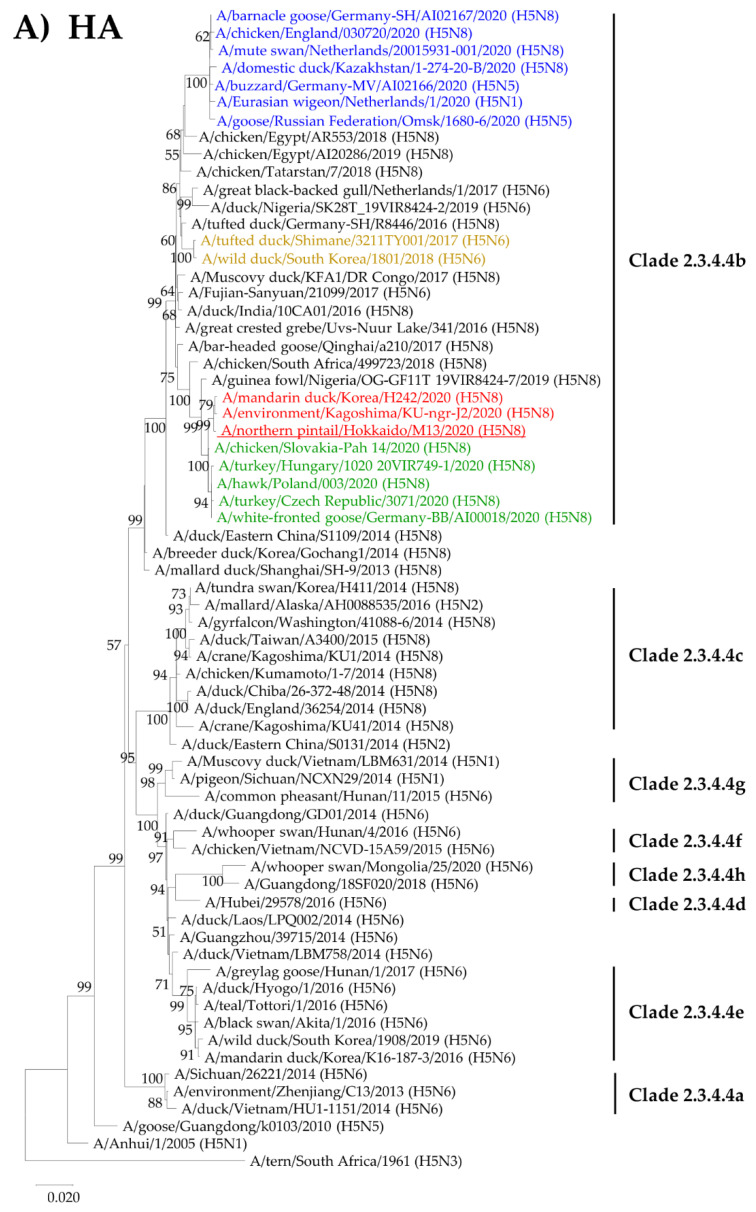

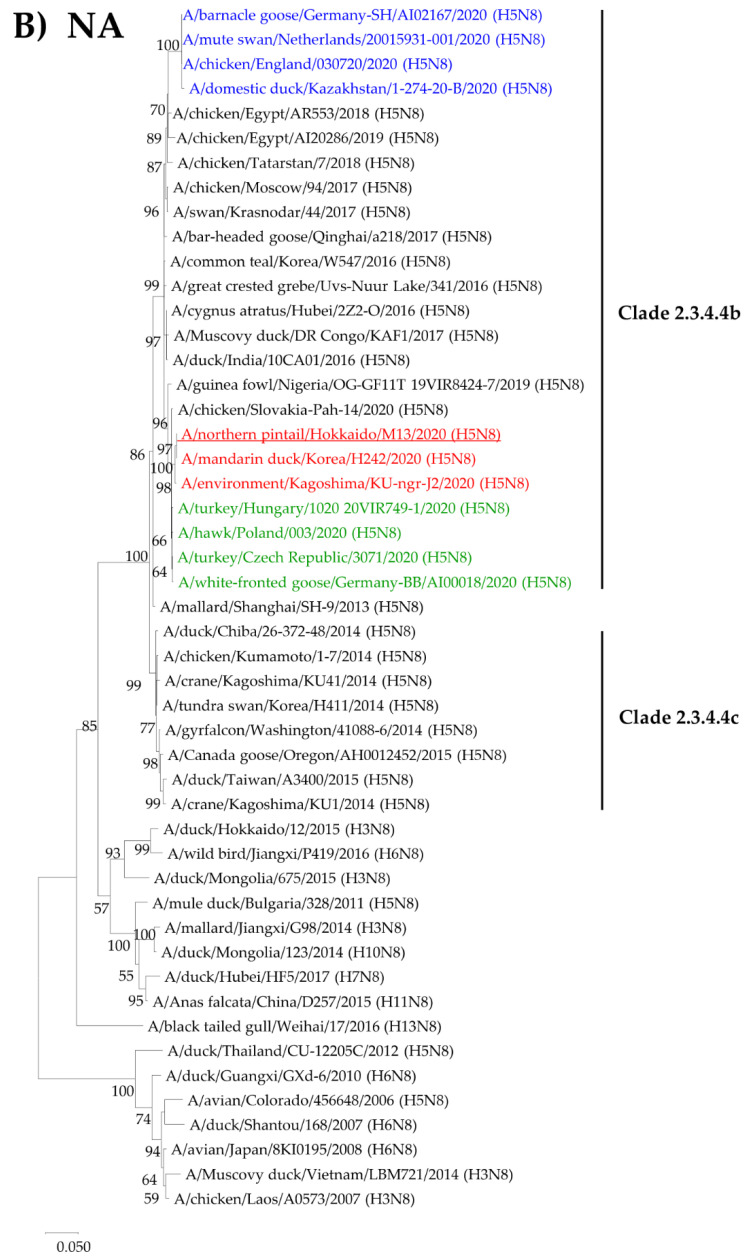
Phylogenetic tree analysis on the hemagglutinin (HA) and neuraminidase (NA) gene segments. Phylogenetic tree analysis was based on (**A**) the HA gene segment containing a total of 67 H5 clade 2.3.4.4, other clades, and outgroups, and (**B**) the NA gene segment containing a total of 49 N8 NA, including highly and low pathogenic avian influenza viruses. The red-colored strain indicates H5N8 high pathogenicity avian influenza viruses (HPAIVs) isolated in Japan and Korea, 2000 of which A/northern pintail/Hokkaido/M13/2020 (H5M8), an isolate in Hokkaido, 2020 is underlined. The green-, yellow-, and blue-colored strains indicate the H5N8 HPAIVs isolated in Europe in the winter of 2019-2020, H5N6 HPAIVs isolated in Japan and Korea in the winter season of 2017–2018, and H5Nx HPAIVs isolated in Europe in October and November, 2020, respectively. The numbers below or above the node indicate bootstrap values greater than 60%.

The full-length nucleotide sequences for all eight gene segments of NP/Hok/20 were analyzed with those of other H5 HPAIVs and some N8 LPAIVs for a phylogenetic analysis of the NA (Figure 2 and Figure S1). All eight gene segments of NP/Hok/20 were completely sequenced and submitted to GenBank (accession numbers: MW228062-MW228069) and were confirmed to be almost identical to strains isolated in Korea, October 2020, or isolated in the southern part of Japan (Kagoshima), November 2020. The phylogenetic tree of the HA segment indicated that the HAs of NP/Hok/20 and the Korean strain were classified in clade 2.3.4.4b and further classified into the same cluster of European isolates in the winter season of 2019−2020, including A/chicken/Slovakia-Pah 14/2020 (H5N8), with which NP/Hok/20 showed the third highest homology (Figure 2A). Within 2.3.4.4b, on the other hand, the cluster containing NP/Hok/20 was apparently distinct both from the H5N6 isolates of eastern Asia and Europe in the winter season of 2017–2018, as well as H5Nx Europe strains isolated in October and November 2020. The phylogenetic tree of other gene segments except N8 NA indicated similar results as that of H5 HA; NP/Hok/20 was classified into the same cluster of European isolates in 2020 (Figure S1). In the phylogenetic tree of the NA gene segment, the NA of NP/Hok/20 was classified into the cluster of H5N8 clade 2.3.4.4b strains isolated in Europe in the winter season of 2019–2020, although it was distinct from the cluster of clade 2.3.4.4c (Figure 2B).

**Figure 3:**
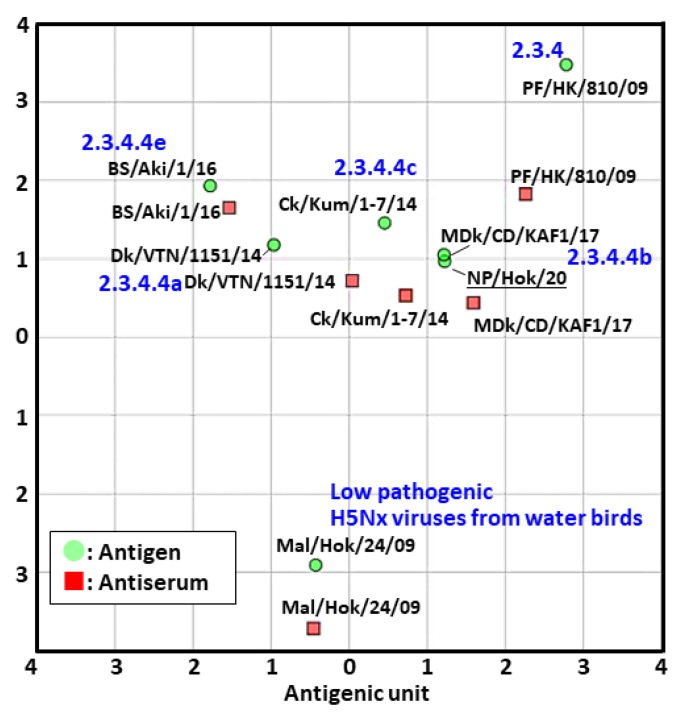
An antigenic map based on the cross-hemagglutination inhibition (HI) tests on H5 clade 2.3.4.4 viruses and the sera of different genetic groups. The antigenic relationship among H5 clade 2.3.4.4 viruses, including NP/Hok/20, was visualized using antigenic cartography based on the results of the cross-HI tests, as indicated in Table S1. In an antigenic map, both vertical and horizontal axes represent the antigenic distance, and the spacing between grid lines represents a distance of 1 antigenic-unit distance, which corresponds to a 2-fold dilution in the HI assay.

The antigenicity of NP/Hok/20 was characterized and compared with that of other groups of clade 2.3.4.4, using an HI test (Table S1). The HI titer of NP/Hok/20 against the antisera of Ck/Kum/1-7/14, MDk/CD/KAF1/17, Dk/VTN/1151/14, and PF/HK/810/09 had the same antigenicity as the HI titers of each homologous antigen and antiserum combination. On the other hand, NP/Hok/20 did not react well to the antisera of BS/Aki/1/16 and Mal/Hok/24/09. The antigenic cartograph developed using the results of the cross-HI tests of Table S1 demonstrated that the clade 2.3.4.4b, including NP/Hok/20, was antigenically close to clade 2.3.4.4c (Figure 3). The isolates of clade 2.3.4.4b would partially react to clade 2.3.4.4a, but were more than two antigenic units away, which were estimated to be the equivalent of a 4-fold HI difference from that of clade 2.3.4.4e. 

## 4. Discussion

In October 2020, an HPAIV, A/northern pintail/Hokkaido/M13/2020 (H5N8) was isolated from a fecal sample of the migratory bird in the northern part of Japan, and a series of HPAI outbreaks in poultry farms have been confirmed in Japan since November 2020 [26]. The results of the sequence analysis indicated that H5 HA of NP/Hok/20 was classified into clade 2.3.4.4b, but into a separate cluster of isolates in eastern Asia during the winter season of 2017–2018. Moreover, the high sequence homology of NP/Hok/20 to the isolates in Europe during the winter season of 2019–2020 implies that NP/Hok/20 was delivered from Europe via bird migration, rather than as a product of genetic reassortment between the HPAI H5 clade 2.3.4.4b strain, which was potentially maintained in East Asia, and other local LPAIVs. Given that dissemination of identical viruses in two far-distinct regions was confirmed, as well as genetically distinct HPAIVs from NP/Hok/20 being prevalent in Europe at the end of 2020, several kinds of highly contagious viruses could have already been perpetuated in northern territories, like Siberia, though this assumption should be highly influenced by the isolation of a virus possessing similar genetic characteristics as NP/Hok/20 in there. It has been understood that influenza viruses perpetuate in aquatic birds including ducks in northern territories like Siberia or Alaska where they usually nest in summer [27,28]. Eastern Asia has been a hot spot for HPAIV outbreaks caused by H5 HPAIV infection for more than 20 years, since most of the new lineage strains and genetic reassortments were produced within or near this region. Japan, like other nations, has tolerated the introduction of the H5 clade 2.3.4.4 by bird migration [29,30,31]. The waterfowl migration pathways satellite-tracked between eastern Asia and eastern Russia support the assumption of HPAIV introduction from eastern Siberia in the fall−winter season and from eastern Asia in the winter−spring season [32,33]. Moreover, a satellite-tracked migration investigation could not confirm the direct migration between central Asia and Hokkaido, Japan, but could verify migration between the eastern part of Hokkaido Japan and the Kamchatka Peninsula [33]. It may indicate that contagious pathogens that were originally isolated in Europe could be transferred via the northern territory to eastern Asia, and could also be carried to the northern part of North America. These speculations would warn us of the likely perpetuation of HPAIV in the north and continuous invasion of HPAIV through bird migration to the south.

Antigenicity variance among different HA lineages is well known. The antigenicity of the African isolate in clade 2.3.4.4b was neither different from the isolate in clade 2.3.4.4a nor that in 4c, whereas a moderate difference observed via antigenic cartography was confirmed between isolates in clades 2.3.4.4b and 4e [34]. The results obtained in the present study are similar, and the antigenicity of between two clade 2.3.4.4b isolates, one from the Democratic Republic of Congo and one from Japan, would almost be the same (Figure 2). On the other hand, expansion of the clusters, including European and Japanese isolates in 2020, from the clusters of past isolates in the H5 HA phylogenetic tree reveals another concern. This finding implied that further genetic variants were selected from strains currently circulating among migratory birds. Further investigation on the H5 clade 2.3.4.4 is required to monitor the potential genetical and antigenic changes. 

An HPAIV H5N8 clade 2.3.4.4b virus was isolated from the fecal sample of migratory bird in Hokkaido, Japan, in October 2020, and was almost identical to European isolates in the winter season of 2019–2020 but was distinct from the European isolates in the end of 2020. As the perpetuation of contagious viruses in northern territories is likely, intensive preparedness for their invasion should be implemented. The possibility of intercontinental HPAIV transportation could admonish us to strengthen the preparedness for its invasion. Enforcement of biosecurity measures on poultry farms and of the exclusion of wild birds from the vicinity, and the monitoring of avian influenza virus circulation in both domestic poultry and wild migratory birds, even in previously unaffected areas, are essential to minimize HPAI threats. Indeed, in Japan, between 30 October and 8 December 2020, a total of 19 poultry outbreaks and eight wild bird cases of HPAI were reported in six and three prefectures, respectively [35]. In parallel, strengthening the function of the global network for related influenza viruses is an urgent agenda so that the genetic and antigenic properties of H5N8 isolates can be used to develop a more effective control strategies against variable strains of H5N8 clade 2.3.4.4.

## Supplementary Materials

The following are available online at https://www.mdpi.com/1999-4915/12/12/1439/s1, Figure S1. Phylogenetic tree analysis on the internal gene segments, Table S1. Cross-reactivity of H5 Highly pathogenic avian influenza virus clade 2.3.4.4.
