# Supplementary material for: Re-Invasion of H5N8 High Pathogenicity Avian Influenza Virus Clade 2.3.4.4b in Hokkaido, Japan, 2020
Source: Viruses. 2020 Dec 14;12(12):1439. doi: 10.3390/v12121439 (PMC7764937; doi:10.3390/v12121439)
Supplement: Supplementary file 1 [file viruses-12-01439-s001.pdf]

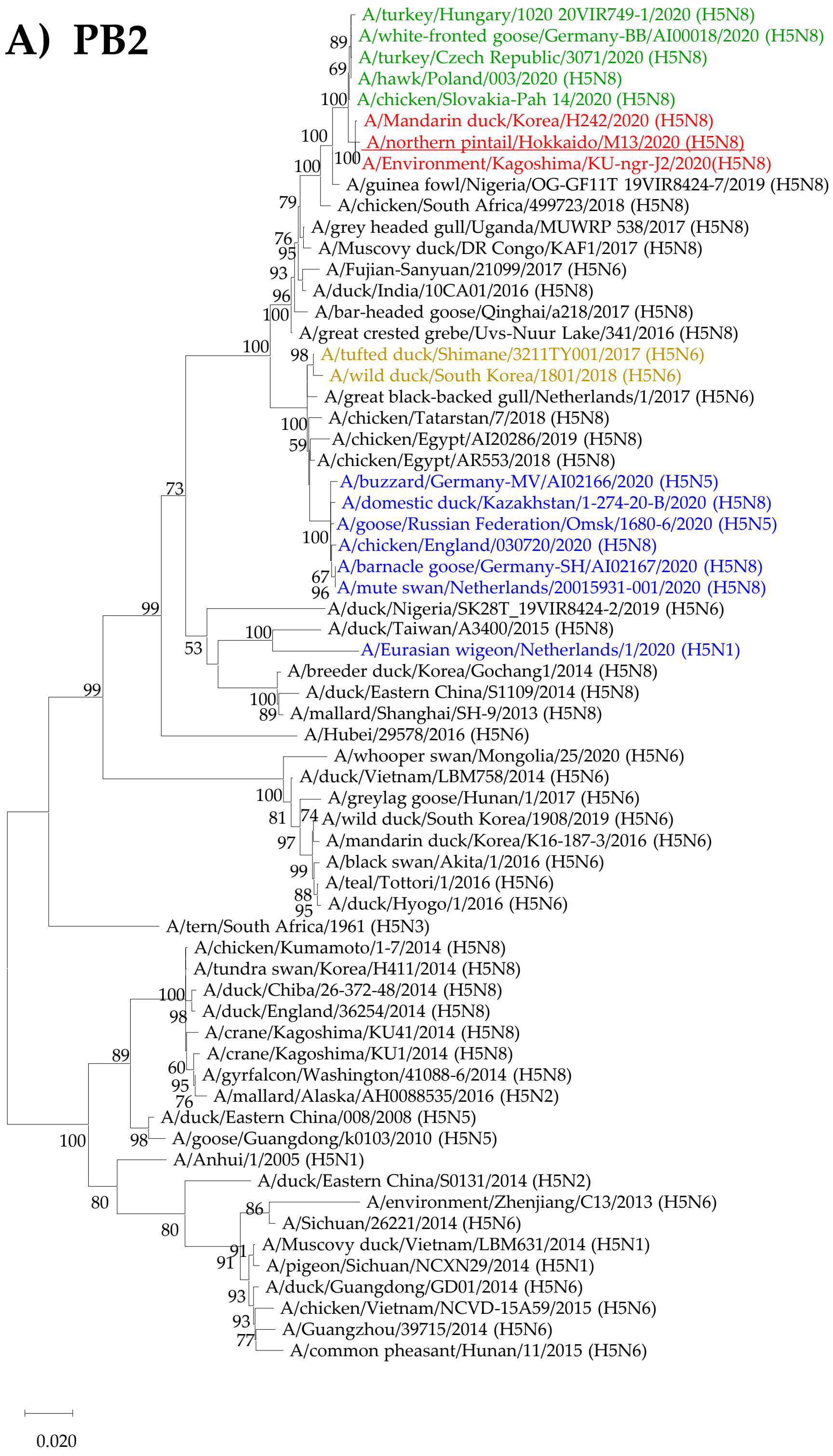

Figure S1. Phylogenetic tree analysis on the internal gene segments. Phylogenetic tree analysis was based on A) the PB2, B) PB1, C) PA, D) NP, E) M and F) NS gene segment containing H5 clade 2.3.4.4, other clades, and outgroups. The red-colored strain indicates H5N8 HPAIV isolated in Japan and Korea, 2020, of which A/northern pintail/Hokkaido/M13/2020 (H5M8) is underlined. The green- , yellow- , and blue-colored strains indicate the H5N8 HPAIVs isolated in Europe in the winter of 2019-2020, H5N6 HPAIVs isolated in Japan and Korea in the winter season of 2017–2018, and H5Nx HPAIVs isolated in Europe in October and November, 2020, respectively. The numbers below or above the node indicate bootstrap values greater than 60%.

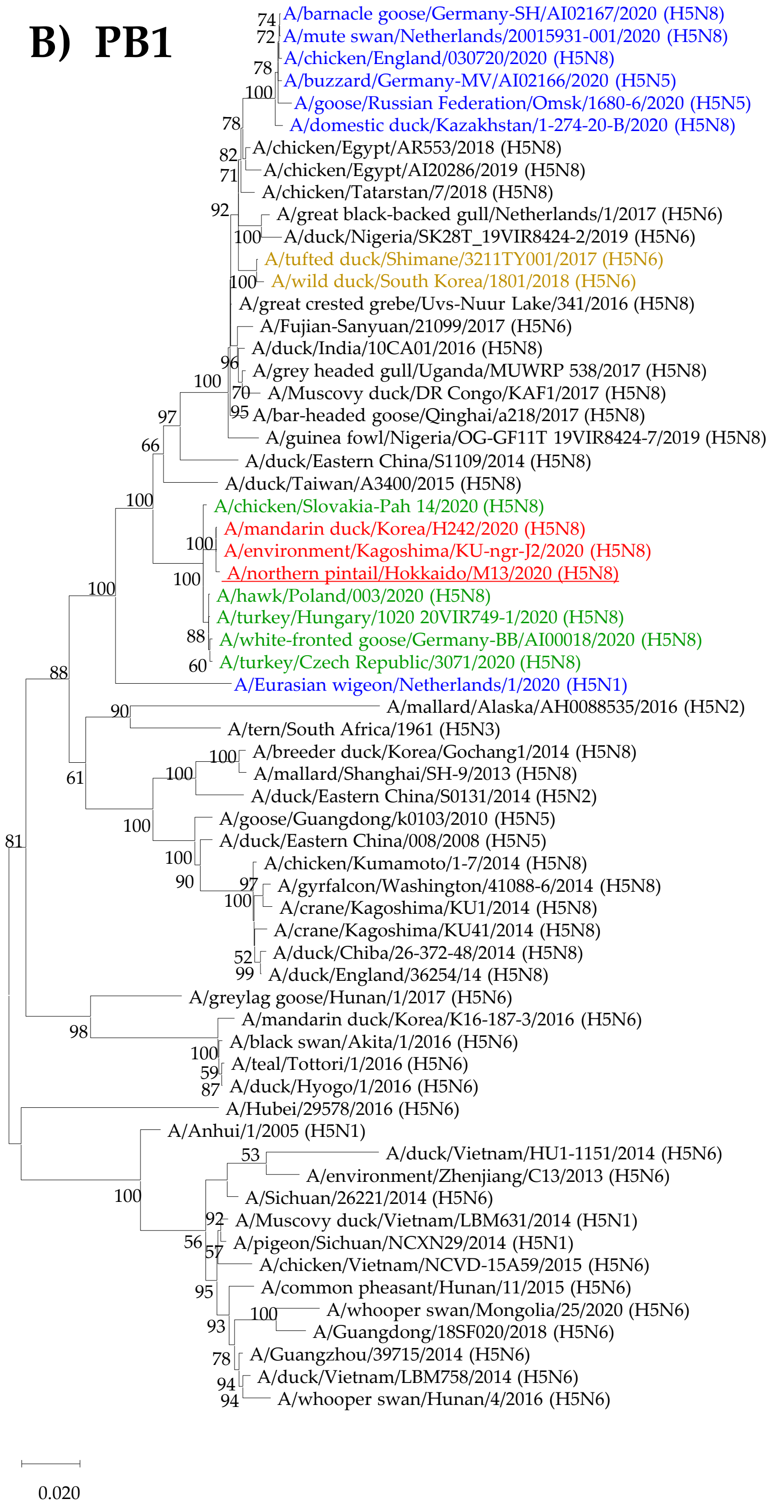

Figure S1. Phylogenetic tree analysis on the internal gene segments. Phylogenetic tree analysis was based on A) the PB2, B) PB1, C) PA, D) NP, E) M and F) NS gene segment containing H5 clade 2.3.4.4, other clades, and outgroups. The red-colored strain indicates H5N8 HPAIV isolated in Japan and Korea, 2020, of which A/northern pintail/Hokkaido/M13/2020 (H5M8) is underlined. The green- , yellow- , and blue-colored strains indicate the H5N8 HPAIVs isolated in Europe in the winter of 2019-2020, H5N6 HPAIVs isolated in Japan and Korea in the winter season of 2017–2018, and H5Nx HPAIVs isolated in Europe in October and November, 2020, respectively. The numbers below or above the node indicate bootstrap values greater than 60%.

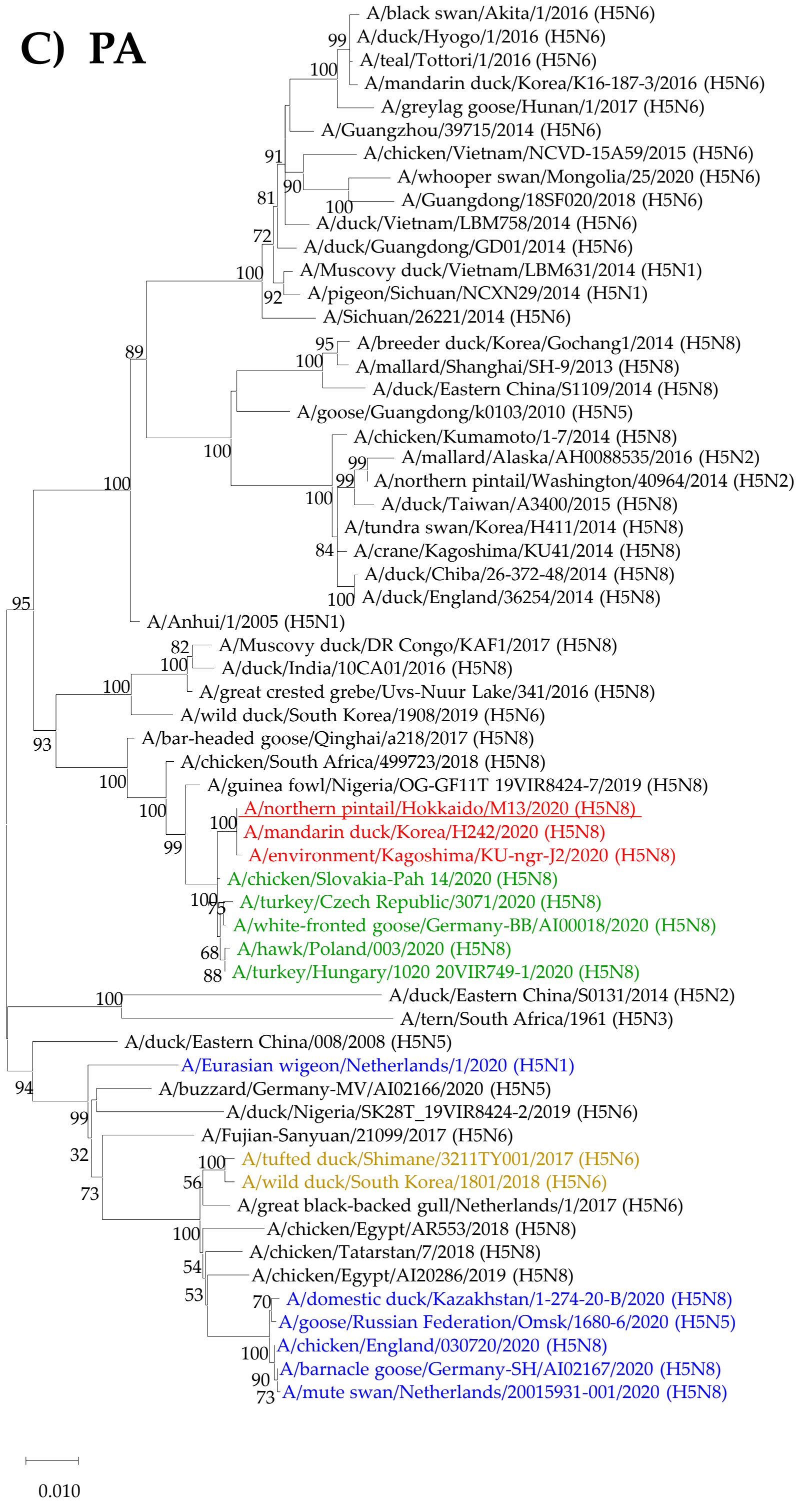

Figure S1. Phylogenetic tree analysis on the internal gene segments. Phylogenetic tree analysis was based on A) the PB2, B) PB1, C) PA, D) NP, E) M and F) NS gene segment containing H5 clade 2.3.4.4, other clades, and outgroups. The red-colored strain indicates H5N8 HPAIV isolated in Japan and Korea, 2020, of which A/northern pintail/Hokkaido/M13/2020 (H5M8) is underlined. The green- , yellow- , and blue-colored strains indicate the H5N8 HPAIVs isolated in Europe in the winter of 2019-2020, H5N6 HPAIVs isolated in Japan and Korea in the winter season of 2017–2018, and H5Nx HPAIVs isolated in Europe in October and November, 2020, respectively. The numbers below or above the node indicate bootstrap values greater than 60%.

D) NP

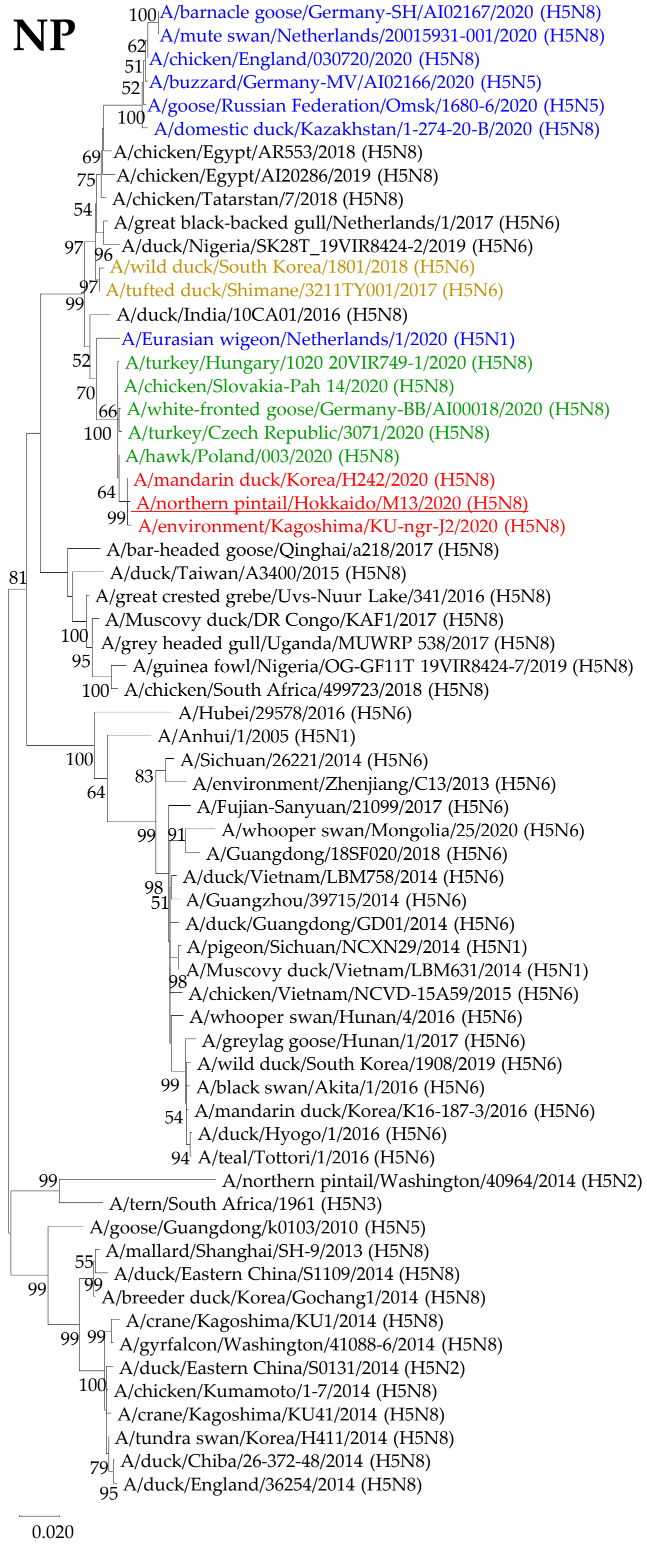

Figure S1. Phylogenetic tree analysis on the internal gene segments. Phylogenetic tree analysis was based on A) the PB2, B) PB1, C) PA, D) NP, E) M and F) NS gene segment containing H5 clade 2.3.4.4, other clades, and outgroups. The red-colored strain indicates H5N8 HPAIV isolated in Japan and Korea, 2020, of which A/northern pintail/Hokkaido/M13/2020 (H5M8) is underlined. The green- , yellow- , and blue-colored strains indicate the H5N8 HPAIVs isolated in Europe in the winter of 2019-2020, H5N6 HPAIVs isolated in Japan and Korea in the winter season of 2017–2018, and H5Nx HPAIVs isolated in Europe in October and November, 2020, respectively. The numbers below or above the node indicate bootstrap values greater than 60%.

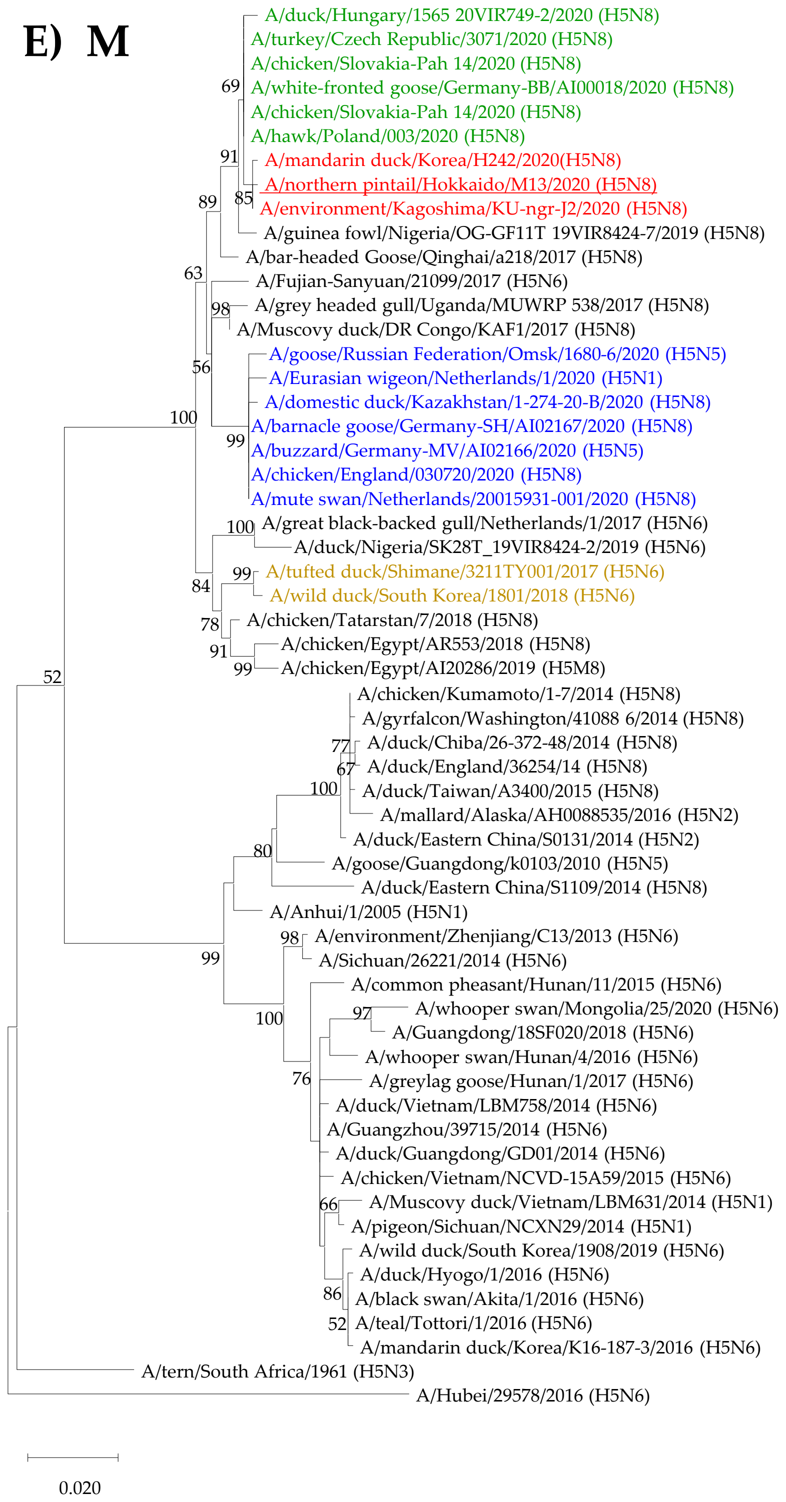

Figure S1. Phylogenetic tree analysis on the internal gene segments. Phylogenetic tree analysis was based on A) the PB2, B) PB1, C) PA, D) NP, E) M and F) NS gene segment containing H5 clade 2.3.4.4, other clades, and outgroups. The red-colored strain indicates H5N8 HPAIV isolated in Japan and Korea, 2020, of which A/northern pintail/Hokkaido/M13/2020 (H5M8) is underlined. The green- , yellow- , and blue-colored strains indicate the H5N8 HPAIVs isolated in Europe in the winter of 2019-2020, H5N6 HPAIVs isolated in Japan and Korea in the winter season of 2017–2018, and H5Nx HPAIVs isolated in Europe in October and November, 2020, respectively. The numbers below or above the node indicate bootstrap values greater than 60%.

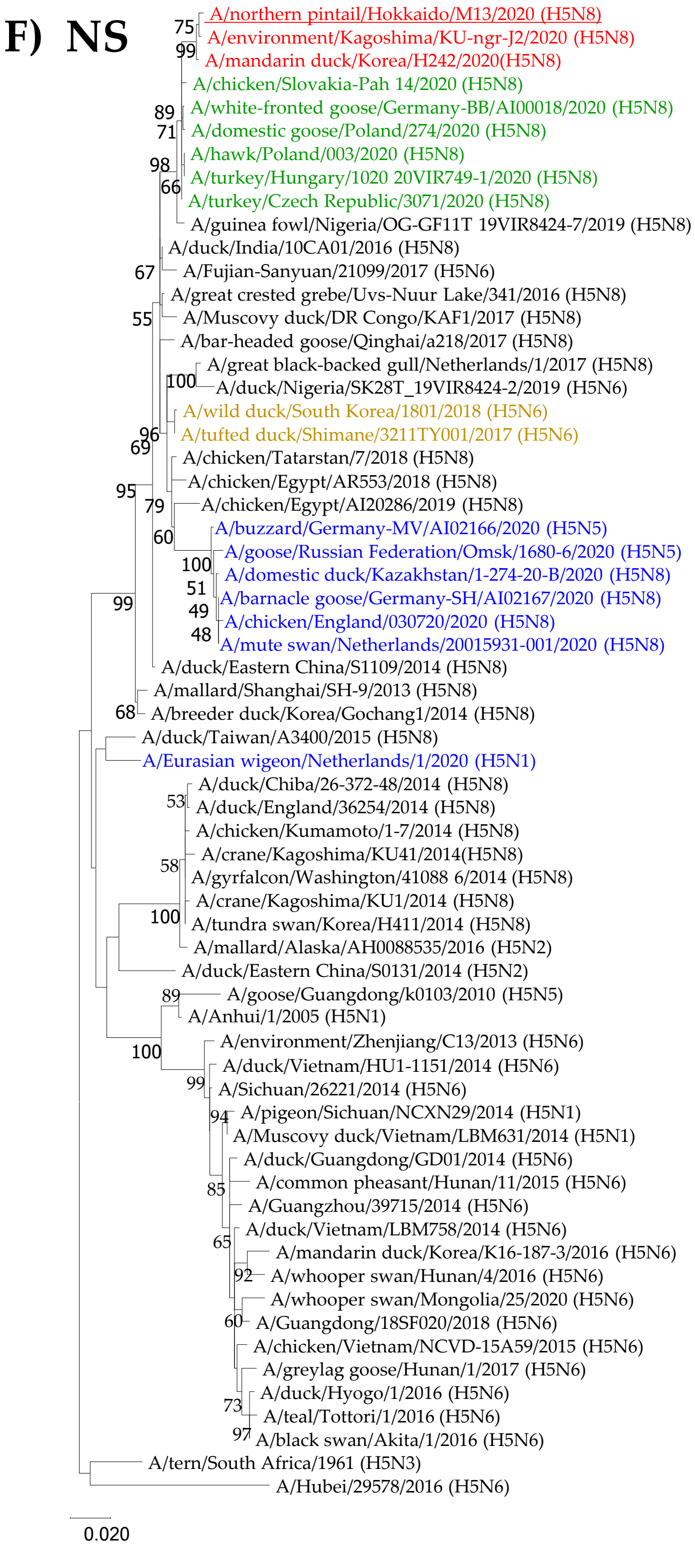

Figure S1. Phylogenetic tree analysis on the internal gene segments. Phylogenetic tree analysis was based on A) the PB2, B) PB1, C) PA, D) NP, E) M and F) NS gene segment containing H5 clade 2.3.4.4, other clades, and outgroups. The red-colored strain indicates H5N8 HPAIV isolated in Japan and Korea, 2020, of which A/northern pintail/Hokkaido/M13/2020 (H5M8) is underlined. The green- , yellow- , and blue-colored strains indicate the H5N8 HPAIVs isolated in Europe in the winter of 2019-2020, H5N6 HPAIVs isolated in Japan and Korea in the winter season of 2017–2018, and H5Nx HPAIVs isolated in Europe in October and November, 2020, respectively. The numbers below or above the node indicate bootstrap values greater than 60%.

Table S1. Cross-reactivity of H5 high pathogenicity avian influenza viruses clade 2.3.4.4

Clade	Virus	Subtype	Antisera					
			Ck/Kum/ 1-7/14	MDk/CD/ KAF1/17	BS/Aki/ 1/16	Dk/VTN/ 1151/14	PF/HK/ 810/09	Mal/Hok/ 24/09
2.3.4.4c	Ck/Kum/1-7/14	H5N8	640	320	40	320	160	20
2.3.4.4b	NP/Hok/20	H5N8	640	1280	40	640	1280	40
2.3.4.4b	MDk/CD/KAF1/17	H5N8	640	1280	80	320	1280	40
2.3.4.4e	BS/Aki/1/16	H5N6	80	80	320	160	80	20
2.3.4.4a	Dk/VTN/1151/14	H5N6	160	160	160	640	80	40
2.3.4	PF/HK/810/09	H5N1	20	80	20	40	1280	<20
-	Mal/Hok/24/09	H5N1	80	80	20	80	40	1280

Underlined numbers indicate homologous titers for each virus and the corresponding antiserum.
